# Supplementary material for: Venous Thromboembolism in Patients with Neuroendocrine Neoplasms: A Systematic Review of Incidence, Types, and Clinical Outcomes
Source: Cancers (Basel). 2025 Jan 10;17(2):212. doi: 10.3390/cancers17020212 (PMC11763766; doi:10.3390/cancers17020212)
Supplement: Supplementary file 1 [file cancers-17-00212-s001.zip › cancers-3401007-supplementary.pdf]

Search Strategy

PubMed

- Search Query:

```
("neuroendocrine neoplasms"[MeSH Terms] OR "neuroendocrine
tumors"[Title/Abstract] OR "neuroendocrine carcinoma"[Title/Abstract])
AND
("thromboembolism"[MeSH Terms] OR "thrombosis"[MeSH Terms] OR "deep vein
thrombosis"[Title/Abstract] OR "pulmonary embolism"[Title/Abstract])
```

- Filters Applied:
    - Language: English
    - Species: Humans

Scopus

- Search Query:

```
TITLE-ABS-KEY("neuroendocrine neoplasms" OR "neuroendocrine tumors" OR
"neuroendocrine carcinoma")
AND
TITLE-ABS-KEY("thromboembolism" OR "thrombosis" OR "deep vein thrombosis"
OR "pulmonary embolism")
```

- Filters Applied:
    - Language: English
    - Document Type: Article, Review
    - Human Studies

Embase

- Search Query:

```
bash
Copia codice
('neuroendocrine neoplasms'/exp OR 'neuroendocrine tumors'/exp OR
'neuroendocrine carcinoma'/exp)
AND
('thromboembolism'/exp OR 'thrombosis'/exp OR 'deep vein thrombosis'/exp
OR 'pulmonary embolism'/exp)
```

- Filters Applied:
    - Language: English
    - Humans

Search Terms and Variations Used Across Databases

Category	Search Terms
**Neuroendocrine Neoplasms**	"neuroendocrine neoplasms", "neuroendocrine tumors", "neuroendocrine carcinoma"

Category	Search Terms
**Thromboembolism**	"thromboembolism", "thrombosis", "deep vein thrombosis", "pulmonary embolism"
**Synonyms/Alternates**	Related terms and synonyms for the above categories, including variations like "NETs", "NECs", etc.

Search Notes

1. The search strategy included both MeSH terms (PubMed) and Emtree terms (Embase) where applicable, alongside free-text terms.
2. Boolean operators (`AND`, `OR`) and truncation symbols (`*`) were used where necessary to broaden or refine the search.
3. No date restrictions were applied to ensure comprehensive coverage from database inception to the present.

Date of Last Search

18/11/2024
